# Improving the Health Forecasting Alert System for Cold Weather and Heat-Waves In England: A Proof-of-Concept Using Temperature-Mortality Relationships

**DOI:** 10.1371/journal.pone.0137804

**Published:** 2015-10-02

**Authors:** Giacomo Masato, Angie Bone, Andrew Charlton-Perez, Sean Cavany, Robert Neal, Rutger Dankers, Helen Dacre, Katie Carmichael, Virginia Murray

**Affiliations:** 1 University of Reading, Meteorology Dept., Earley Gate, Reading, United Kingdom; 2 Public Health England, Dept. of Health, Waterloo Rd, London, United Kingdom; 3 Met Office, FitzRoy Road, Exeter, United Kingdom; The Ohio State University, UNITED STATES

## Abstract

**Objectives:**

In this study a prototype of a new health forecasting alert system is developed, which is aligned to the approach used in the Met Office’s (MO) National Severe Weather Warning Service (NSWWS). This is in order to improve information available to responders in the health and social care system by linking temperatures more directly to risks of mortality, and developing a system more coherent with other weather alerts. The prototype is compared to the current system in the Cold Weather and Heatwave plans via a case-study approach to verify its potential advantages and shortcomings.

**Method:**

The prototype health forecasting alert system introduces an “impact vs likelihood matrix” for the health impacts of hot and cold temperatures which is similar to those used operationally for other weather hazards as part of the NSWWS. The impact axis of this matrix is based on existing epidemiological evidence, which shows an increasing relative risk of death at extremes of outdoor temperature beyond a threshold which can be identified epidemiologically. The likelihood axis is based on a probability measure associated with the temperature forecast. The new method is tested for two case studies (one during summer 2013, one during winter 2013), and compared to the performance of the current alert system.

**Conclusions:**

The prototype shows some clear improvements over the current alert system. It allows for a much greater degree of flexibility, provides more detailed regional information about the health risks associated with periods of extreme temperatures, and is more coherent with other weather alerts which may make it easier for front line responders to use. It will require validation and engagement with stakeholders before it can be considered for use.

## Introduction

It is widely recognised that human health is affected by weather conditions, and in particular by temperature variations and its extremes [1]. Excess deaths occur during both cold weather [2; 3] and heatwave [4; 5] conditions, with particular impacts experienced by the very young, very old and those with chronic diseases such as cardiovascular and respiratory disease. The UK is no exception, and excess mortality has been reported from both heatwaves and cold weather. Excess winter deaths are observed each year in England; there are on average 25,000 excess deaths in winter in England and Wales, a much higher figure compared to the non-winter months [6; 7]. Excess mortality has been recorded during several heatwaves in England, with the greatest number of excess deaths occurring during the heatwave of 2003, where an estimated 2000 excess deaths compared to the same period during the previous five years [8].

A key part of reducing the vulnerability of the population to extreme temperature conditions is to provide all agencies with responsibility for the care of those vulnerable people with timely, accurate and useful warnings of extreme temperatures. Given the ageing population in many developed countries [9] and the likely increase in extreme weather events during the 21^st^ century [10; 11], heat-related mortality is predicted to increase substantially and cold-related mortality to reduce to a lesser degree [12] although cold temperature extremes may still occur in a warming climate as the result of natural variability [13].

There is no universally agreed definition of a heatwave or cold spell, and no single method for developing warning systems for health. There is a wide diversity of approaches for severe heat, ranging from national top-down systems (often used in European countries such as England and France) to more decentralised, city-wide approaches as used in Australia and North America. Some use single-parameter approaches, others use more complex heat-budget or synoptic models [14]. A variety of thresholds and durations are used for criteria to issue a warning. There is a similar diversity of approaches for cold weather warnings.

As is the case in other countries, in England there are a large number of potential end-users of an alert system who would need to act upon the issued alerts. The system should build on the wide-ranging and very successful method for issuing advice during periods of extreme weather developed by Public Health England (PHE). A full discussion of how this advice is currently issued and acted upon can be found in the annual Heatwave [15] and Cold Weather [16] plans issued by PHE. Typically, in circumstances of extreme weather, advice is cascaded through various governmental bodies through to end-users as shown in Fig 2.4 of the Heatwave plan and Figure 2.5 of the Cold Weather plan. To provide some context for the present study, see S1 Table, which lists all potential end-users of the system and their primary point of contact for extreme weather alerts.

Refining this warning system to make best use of existing knowledge in both public health and meteorology is extremely important. In England, the alert system currently in use for the heatwave and cold weather plans is based on five alert levels (see for example [15; 16]).

- “Long-term planning” (level 0) which is operational all year, and relates to longer term actions to reduce vulnerability to temperature extremes such as housing
- “Preparedness” (green, level 1) which lasts for a fixed time period (four months during the summer and winter seasons),
- “Alert and readiness” (yellow, level 2) which is issued when there is a 60% confidence that a temperature forecast will exceed (or for the cold plan, drop below) a given trigger temperature for action. For cold weather alerts, there is a single trigger temperature for all regions of the UK (daily mean temperatures of 2°C), whereas in the trigger for heatwave alerts varies across the nine government regions of England. The heatwave action trigger has a persistence criterion (temperatures must exceed the threshold for at least two days) whereas the cold weather action trigger temperature does not. In addition, the level 2 cold alert can be issued if there is a forecast of widespread ice and snow.
- “Action” (amber, level 3) which is used when extreme weather is occurring, and
- “Major incident–emergency response” (red, level 4) which is only issued after central government declares a *major incident*.

**Fig 1:**
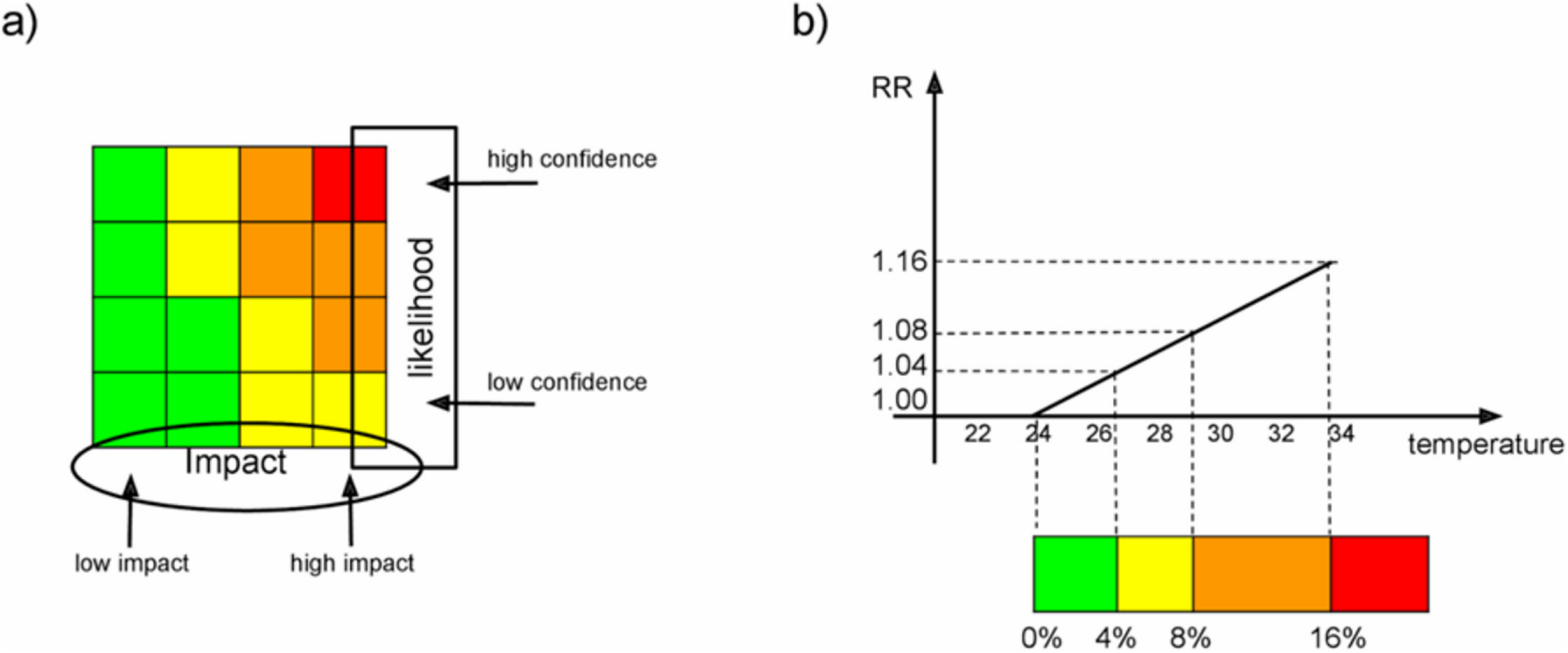
a) Schematic of the impact vs likelihood matrix, derived from the NSWWS. The alert code depends on both the uncertainty of the forecast (along the columns) and the strength of the impact (along the rows); b) Schematic showing the relation between the RR (excess deaths) and the temperature during summer (in degrees C). The temperature range is adjusted to reproduce a RR between 1.0 and 1.16 by using the linear relationship.

There are three major challenges with the existing system. Firstly, although the cold weather and heatwave alert systems are broadly similar, they are not consistent with the current system used for the National Severe Weather Warning Service (NSWWS) of the Met Office (MO) [17], potentially leading to confusing or contradictory messages. The NSWWS provides warnings about rain, wind, fog, snow and ice, but not temperature extremes and uses a traffic light system based on a matrix of predicted impact and likelihood (Fig 1A), thus taking a different approach to the ‘get ready, go’ approach of the heatwave and cold weather alert services.

Secondly, heatwave and cold weather alerts are issued at Met Office regional level (9 regions) but this does not reflect differences in temperature within a region which may influence the risk to health and therefore the appropriateness of actions to be taken by frontline responders. Provisions of alerts at a more refined geographical level have been frequent requests from end-users of the system (for example [18; 19]).

Lastly, the action trigger temperatures used in the current heatwave and cold weather alert services are not clearly linked to the available epidemiological evidence of impact on health. This is particularly so for the 2°C trigger of the cold weather alerts, which was a pragmatic choice to deliver a certain number of alerts each winter [20]. The regional temperature triggers used in the heatwave plan are more closely linked to the available epidemiology and have been shown to be consistent with odds ratios of mortality of above 1.15–1.20 (a 15–20% increased risk, see [15], page 39). However, neither system reflects the approximately linear increase in relative risk (RR) of death once temperature moves just beyond a threshold which can be defined epidemiologically (see for example [21]). Moreover, the existing 60% risk of a forecast hot or cold weather event remains a rather crude probabilistic measure of the likelihood of an extreme event, in particular if compared with the newest techniques used by the Numerical Weather Prediction centres to assess forecast uncertainty. These are now based on an ensemble prediction system (EPS) which contains a set of different forecasts (or ‘members’) initiated with different initial conditions, chosen to represent the spread of uncertainty of the atmosphere (given by its chaotic nature).

For all the above reasons the need of an improved system has recently sparked. The prototype should therefore address two main points. The first is the possibility to issue alerts that are not merely based on meteorological factors (such as temperature), but rather on the impact that such factors may have on human health, such as mortality or morbidity. The second is the potential to deliver the alerts at an improved geographical scale, allowing the end-users to take sensitive decisions at a local level if need be. The large improvement of weather forecasts, along with the increased knowledge on the relationship between weather and human health, are paramount for the design of a new prototype system, whose properties will be explained in the next section.

## Method

### Prototype system

A crucial point in the design criteria for the proposed new method was to make it consistent with the NSWWS system [17], where risk is the product of a measure of the likely impact of the hazard and the uncertainty of the underlying weather forecast (e.g. [22]). Thus:

1. a refined “local approach” to be applied to both the cold weather and heatwave alerts;
2. an impact range that depends on epidemiological evidence (i.e. mortality-temperature relationship);
3. an uncertainty range that depends on the properties of the forecast (e.g., the forecast lead time, which is the length of time between the issue of a forecast and the occurrence of the phenomenon that is predicted).

In this study we will mainly focus on points 1 and 2, which are more relevant from a public health point of view, and discuss possible ways of implementing point 3, which depends on the operational weather forecasting system in use and which is likely to change as this system is updated.

Fig 1A shows a schematic of the concept, whereby the alert level is associated with *a* measure of *impact* and *likelihood*. The latter (y-axis in the matrix of Fig 1A) is given by the confidence of the temperature forecast. In this example this is related to the amount of time between the forecast being issued and when the event is due to occur. This is a rather crude approach, which is based on the assumption that a forecast accuracy is much greater when closer to the event occurrence. At the end of the *Results Session* an example will be provided whereby the likelihood of an extreme event is calculated more objectively via the EPS approach in use at the Met Office (it is worth noting that at present uncertainties in the weather forecast are routinely being assessed using EPS giving probabilistic forecasts—e.g. [23]).

The *impact* (x-axis in the matrix of Fig 1A) *is* based on the severity of the predicted mortality RR. Fig 1B shows in greater detail how the risk is derived from existing epidemiological evidence linking mortality RR with temperature. A RR of 1 implies no excess deaths and a RR of 1.04 implies a 4% increase in deaths. For this example, a yellow alert occurs when the RR lies between 1.04 and 1.08. It is noted that this example is only illustrative, as the appropriate relative risk of death thresholds at which yellow, amber and red warnings for heat and cold are issued will need to be determined in discussion with stakeholders. The colour code in the example follows that of the NSWWS, to make the two systems as consistent as possible.

This approach allows us to derive a mean temperature range over which this enhanced risk would occur, based on the relationship between RR of death and temperature. The same approach can be used for both extreme heat and cold and it is possible to incorporate regional variation in temperature-mortality relationships, as long as the epidemiological data are available. Although other systems make use of more complex biometeorological indices, the reason for choosing surface mean temperature is twofold. Firstly, its relationship with mortality has already been clearly demonstrated (see below). Secondly, this proof-of-concept system has been created to be as simple as possible, using the most basic meteorological variable, with known predictability characteristics and that is known to impact human health. The novelty of the proof-of-concept system is the incorporation of the vulnerability and likelihood dimensions of risk into one system. The proof-of-concept is sufficiently flexible that should it be necessary to incorporate more complex epidemiological information into a future version, this could be done at minimal cost.

For England, RR-temperature relationships are derived from [21] and [12]. [21] analysed the relationship between the RR and temperature in the summer period for the nine government regions of England (plus Wales), using daily counts of all-cause mortality and measures of ambient temperature between 1993 and 2006. Individual death record data were obtained from the Office for National Statistics, including date of death and postcode of residence at time of death, while the temperature values were downloaded from the British Atmospheric Data Centre for all available monitoring station data. For each region a linear model has been fitted with a good approximation (particularly for a RR<1.2). Using the linear model, we can derive a temperature range for each of the nine regions, which is associated with a fixed interval in excess deaths. For the illustrative purpose of this study, we have derived temperature intervals for four RR bands (1.0–1.04, 1.04–1.08, 1.08–1.16, greater than 1.16), but the thresholds can be easily modified according to operational needs (for example to balance the need to warn the public and responders with the risk of alert fatigue).

It is noted here that there is limited evidence of an increase RR of death as a function of event persistence (e.g. [24]), although some studies have used two day mean temperatures as the exposure variable in modelling temperature mortality relationships (e.g. [21]). For the sake of simplicity, the prototype system was developed and tested using single day mean temperatures. The previously referred case-study (summer 2014) at the end of the *Results Session* introduces a persistence criterion based on the mean of the maximum temperatures on each pair of consecutive days. If such a criterion is proved to be useful, it could potentially be applied to the winter season as well, where the “lag effect” is well documented in the literature.

Vulnerability to extreme temperature is known to vary across the population, with higher vulnerability in very young and elderly population. The RR used in the proof-of-concept system is not age dependent in common with the existing operational system. As always when designing an operational system there has to be a balance between the complexity and comprehensiveness of the system with its ubiquity and utility for the broadest possible group of end users. Our judgement is that because the system is used for the broad range of stakeholders identified in S1 Table rather than targeted at a particular sector with a skewed age profile the choice of a generic RR applicable to all the population is most appropriate. As above, the design of the proof-of-concept system is sufficiently flexible that adapting this for more highly age differentiated age profiles would be a low-cost adaptation should this be desired.

### Case studies

Two case studies have been considered, both of which occurred during 2013, one during the winter season 2012/13, and one during summer. As the prototype is a proof-of-concept, the case studies provided here are not intended to comprehensively test the new system or to objectively assess its performance against the existing approach, rather they are useful to compare advantages and possible shortcomings of the former. The first guess alerts produced by the prototype will be analysed against the recorded alerts of the current system, to highlight the major differences in the timing and space distribution of the alerts. A particular attention will be given to the way such alerts are provided with the potential new system, where detailed geographical information can be visualised to deliver the results effectively and compellingly.

The first guess alerts for the summer case have been based on the temperature at noon, whereas the first guess alerts for the winter cases are associated with the mean temperature, T ([T(midnight)+T(noon)]/2), in order to be consistent with [21] and [12]. One could certainly make other choices (for example, deciding that the night temperature for summer is equally important), however we would like to reiterate that such decisions are not paramount for the results of this research, as the main focus is to demonstrate that the proof-of-concept is an overall advancement over the current system. We also note that winter temperatures associated with a RR>1 (ranging from 4 to 8°C depending on the region–[12]) are not that infrequent during English winter, and certainly higher than the current 2°C threshold. Considering the winter temperature climatology over the UK, this means it would be quite common to have yellow, amber and red alerts if the thresholds were to be set at the levels used in this example (for example, a RR of 1.16 is associated with temperatures ranging between -2 and 4°C for different regions).

The summer case study uses 1 August 2013, where high maximum temperatures (above 30°C) were recorded over south-eastern and central England. The days before and after the maximum temperatures barely exceeded 25°C over the southern part of the country, the event is therefore associated with a single day spike in high temperatures. The winter case is profoundly different as it represents particularly cold conditions on the 11 and 12 March which occurred during an extremely cold month, which was “the coldest March since 1962 and the equal second-coldest in the series from 1910” [25]. On the 11 and 12 of March, the temperature remained at the freezing level (and below) for the entire day and over almost all of the UK, in particular on the 11th.

## Results

### Summer case: Existing system

In Table 1 the alerts issued with the current health forecasting system at the time of the event are listed, from two days before the event date of 1 of August and until the event. The East and South-East regions were assigned a “level 2” alert on 30^th^ July; on the 1st of August a “level 2” alert was raised for the London area as well. No “level 3” alert was issued over the time period, which could be due to temperature not exceeding the given thresholds, or to the persistence criterion of two consecutive days above the threshold not being met. Overall, the alert is set to a “readiness” level, which requires actions to prepare for an imminent heatwave. Furthermore, the regions alerted were limited to the south-eastern part of the country, with the rest of the country at a level 1 “preparedness” programme.

**Table 1:** Summary of the alerts issued at the end of July 2013 by the current system in use in the Heatwave Plan for England. The regional risk of meeting heatwave action trigger temperatures is in percentage and is shown for each of the 9 NSWWS regions.

Alert level	Issued on	Start Date	NEE	NWE	YH	WM	EM	EE	SEE	LON	SWE
Level 2	30/07/2013	01/08/2013	20	20	30	30	50	60	60	50	30
Level 2	01/08/2013	01/08/2013	20	20	30	30	50	60	60	60	30

### Summer case: Prototype

**Fig 2:**
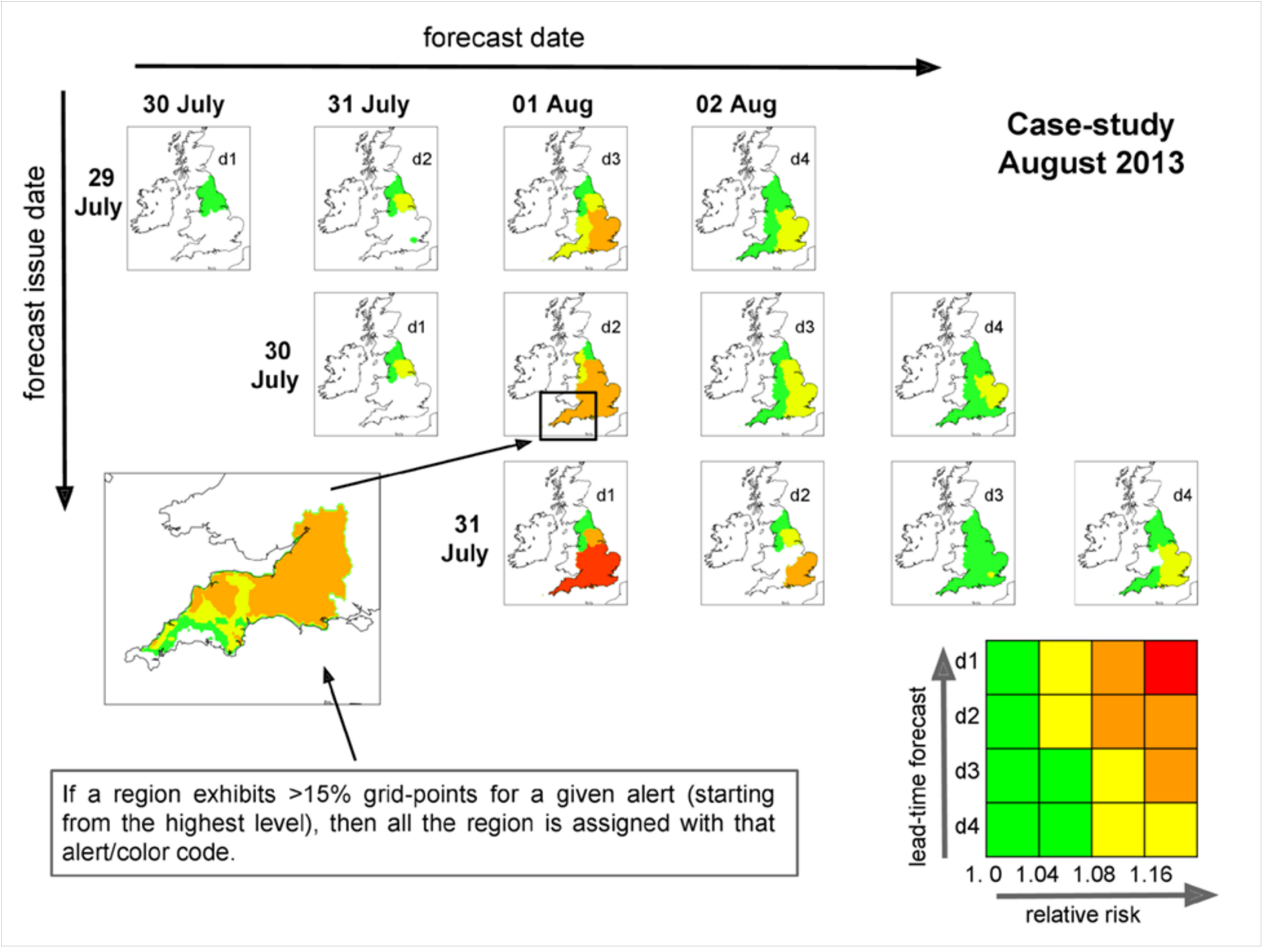
Case study for August 2013. The first guess alerts generated by the prototype system are shown along each row (for the day after up to 4 days ahead). The days are rearranged to be always the same along the columns. The color scheme is explained with the matrix at the bottom right of the figure (where the forecast lead time is used as a proxy for forecast uncertainty). The bottom left panel illustrates an example of a detailed regional alert for the South-West. Scotland, Wales and Northern Ireland are left blank as they do not share the same alert system for health forecasting.

Results from the new method are shown in Fig 2. Four different maps are produced for each forecast, on the 29, 30 and 31 of July. These show the four days ahead of the forecast, and in Fig 2 they are shifted along the forecast day’s time-axis by 1 place each day in order to have the same (forecast) day aligned in each column. The colour scheme is the same as that used in the NSWWS.

The new alert system forecasts a RR risk of death of between 1.08 and 1.16 (amber) three days in advance (forecasts issued on the 29 of July for 1 August) for the entire south-eastern side of the country, including the East Midlands. The amber alert is confirmed the day after (forecasts issued on the 30 July for 1 August) and further extended over the South-West, West Midlands and the Yorkshire. Finally, on the day before the event (forecast issued on 31 July for 1 August), the alert level is raised to its maximum (red, RR of death >1.16) for all of South and Central England, whereas it remains “amber” for Yorkshire. No other red first guess alerts are generated during this period, and a single amber alert is raised for the South-Eastern region on the 2nd of August.

A further advantage of the new approach is the possibility to explore in more detail how the regional alert might vary within region based on detailed temperature information present in the MO forecasts. For example, a Local Resilience Forum (LRF) in the South West might be interested in understanding why the region has been assigned with an amber alert for the forecast issued on the 30 of July. The panel at the bottom left of Fig 2 shows the potential for an end-user to zoom in on any given region to observe the spatial distribution of the alert code. It is evident that most of the South-West is labelled with an amber alert, however the western side of the region has a lower level of risk, with alert levels down to green (or even a complete absence of warnings). The threshold chosen here to label the entire South-West region with the amber alert is set to 15% of its territory. That is, if starting from the highest level, at least 15% of the region is in a given alert category, the entire region will be assigned with that alert level. The authors are aware that such an approach is rather cautious, and could lead to a higher frequency of first guess alerts. However, this is done specifically to test how the new system could have worked, always bearing in mind that the thresholds for issuing region wide alerts are for illustrative purposes and can be easily modified.

Sometimes a given alert code may result from different combinations of impact and likelihood in the matrix (see for example the upper-right corner for the amber first guess alerts). The implementation of the system on a graphical interface will always allow the end-user to view from which point of the matrix, the alert has been generated, for example with a tick mark in the relevant box (as used in [26]). Specifically for this example, the majority of the amber region falls in the matrix box with a RR between 1.08–1.16 for a 2 days lead-time forecast.

### Winter case: Existing system

Table 2 shows the alerts issued with the current health forecasting system during the peak of the cold event in March 2013. A “level 2” warning was issued on the 8 of March, valid from the 9 until the 12 March. A probability greater than 60% of a cold event was forecast for all 9 regions. The alert level was kept at “level 2” until the 10 March, when it was upgraded to “level 3” (severe weather action to be taken) for all regions. The alert was also extended until the 14 March, while the 9^th^ of March remained under a “level 2” alert.

**Table 2:** of the alerts issued during March 2013 by the current system in use in the Heatwave Plan for England. The regional risk of meeting heatwave action trigger temperatures is in percentage and is shown for each of the 9 NSWWS regions.

Alert level	Issued on	Start Date	NEE	NWE	YH	WM	EM	EE	SEE	LON	SWE
Level 2	08/03/2013	09/03/2013	80	80	80	80	80	80	80	70	60
Level 3	10/03/2013	10/03/2013	100	90	100	90	100	100	90	90	90

### Winter case: Prototype

**Fig 3:**
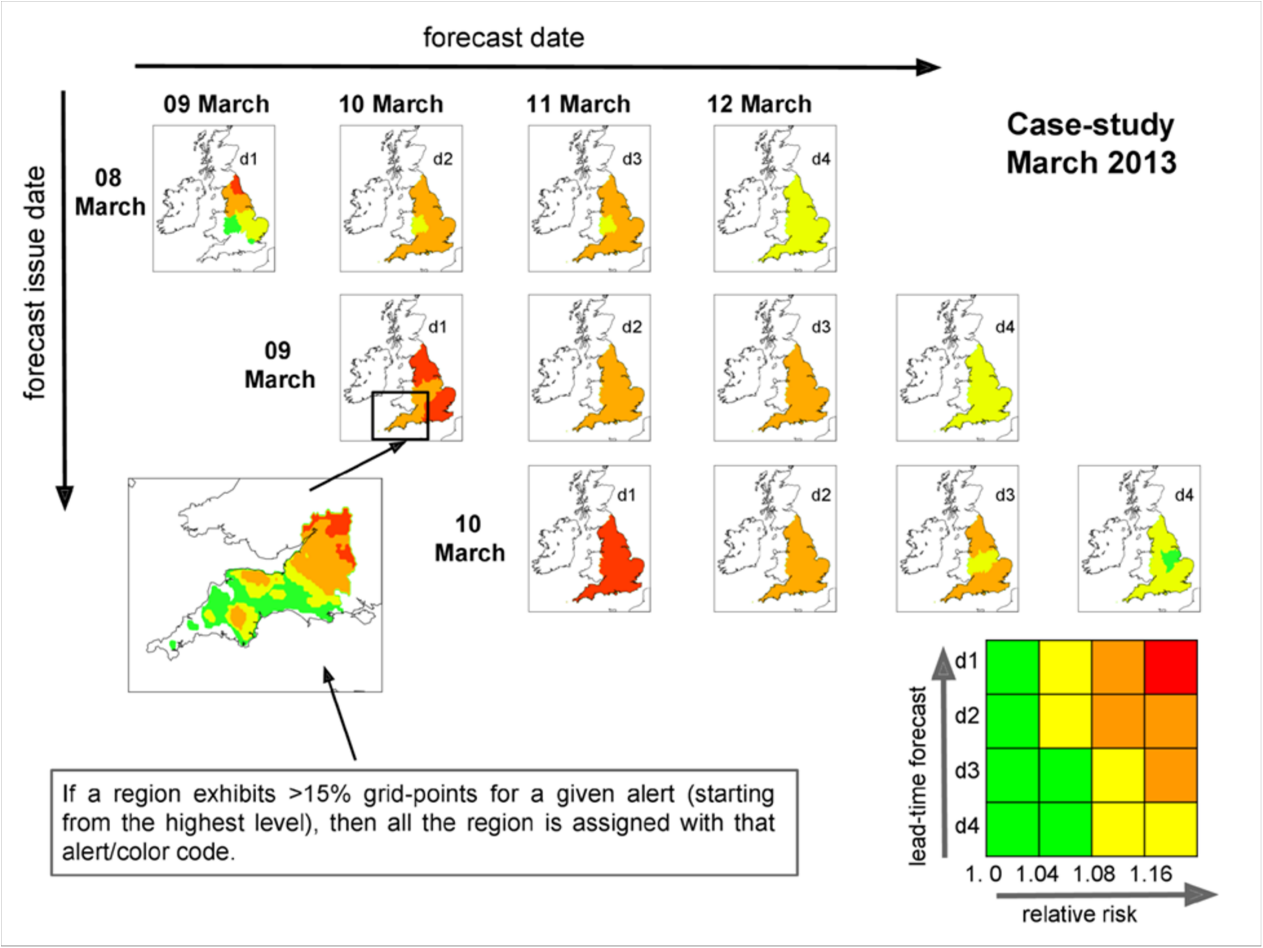
As in Fig 2, but for the case during March 2013.

Fig 3 shows the first guess alerts for the cold case study as issued with the new alert system. As expected, the combination of relatively low thresholds for the RR (e.g. red alerts issued for RR> = 1.16) and the extremely cold period leads to repeated amber and red alerts. Forecasts issued on the 8 and 9 March for the 10, 11 and 12 March indicate the maximum possible alert level up to 4 days (and at least 2 days) before the event occurrence. All regions would issue first guess alerts, giving a clear signal of a potentially highly damaging health impact event. This example also shows clearly the potential for the alert level to increase as time progresses, moving down the columns corresponding to the 11 and 12 of March, which is associated with the increased confidence of the forecast alert as it gets closer to the event date. On the 8th of March, for example, when an amber warning was generated, it was clear that 10 and 11 March would show amber alerts in most regions in the following days.

In this example, for the forecast issued on the 9 March for the 10 March, all possible first guess alerts are displayed for the South West region. Similarly to the summer case, the alerts increase along a west-east direction, with the south-western tip of the region alert-free and the most north-easterly area alerted with a red warning. In this case though, the area associated with the highest alert is below the 15% threshold, so that in the national map the South West region is amber.

### Summer 2014 trial period

**Fig 4:**
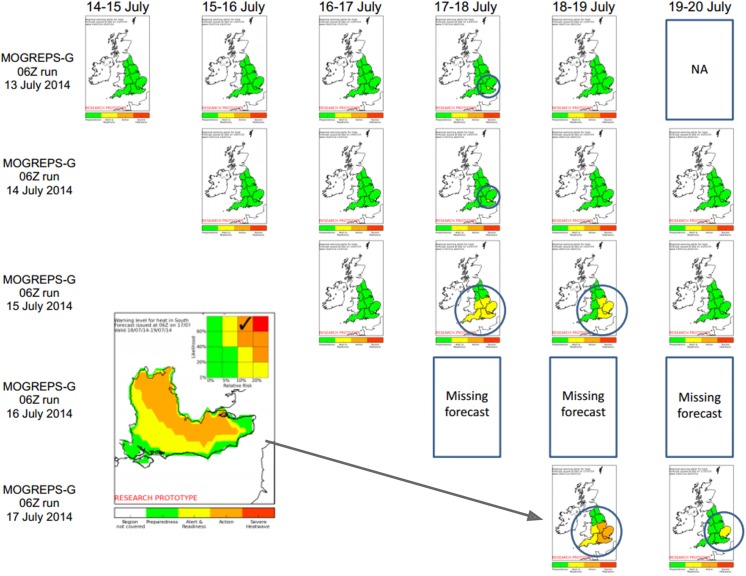
Semi-operational first guess alerts for summer 2014. As in Figs 2 and 3, the forecast dates are along the top, and the issue dates down the side. “Z” refers to Zulu Time as is the same as UTC. Dates with alerts are circled in the figure. The image in the bottom left shows the local forecast for South East England on 18–19 July, issued on 17 July. In the top right of the smaller image one can see the matrix indicating the most severe type of event forecast in that region. Note that forecasts issued on 16 July are missing due to the system going down on that day.

Since July 2014, a prototype first-guess support tool for the proposed new alert system has been running semi-operationally at the Met Office. This prototype is similar to MOGREPS-W (the Met Office Global and Regional Ensemble Prediction System first guess Warnings tool–[17]) and automatically generates daily first guess alerts out to a five day lead time, with updates four times a day at 00, 06, 12 and 18 UTC. Output is displayed on internal web-pages viewable by Met Office and Public Health England staff. Once operational, meteorologists would use this tool to be informed about the likelihood of exceeding alert levels in different regions, and then refine the warnings taking account of other information such as output from high resolution models, current and previous weather conditions, and input from health professionals such as Public Health England and LRFs. This system involves five changes from that discussed above:

1. The likelihood axis of the matrix now uses a measure of probability derived from an ensemble forecast, which in this case is the global component of MOGREPS. The spread of the forecasts from the ensemble members then give an indication of the uncertainty of the forecasts. In this case, probabilities are derived by calculating the fraction of ensemble members which pass a given temperature threshold related to a specified level of RR;
2. A range of impact thresholds are being trialled in order to assess which are the most appropriate;
3. The total lead time of the forecast is now five days rather than four. This is due to using an updated Met Office forecast which itself has a longer lead time;
4. Local first guess alerts are generated alongside an indication of which region of the matrix the forecast lies, to enable different responses to high-impact low-likelihood events versus low-impact high-likelihood events (see Fig 4).
5. The temperature measures used to generate alerts have been amended, based on the measures used in the underlying epidemiological studies. For hot temperatures this involves taking the mean of the maximum temperatures on each pair of consecutive days, and for cold temperatures taking the mean of the maximum and minimum temperatures on each day.

It is relatively easy to apply this impact based first guess warning concept to other probabilistic forecast data sources. Alternatives to MOGREPS-G may include the global medium-range EPS from the European Centre for Medium-Range Weather Forecasts (ECMWF) [27] or the Met Office convection-permitting UK EPS (MOGREPS-UK) [28; 29]. Some of the alternative data sources may provide an even longer lead time to MOGREP-G, but this will typically come at the cost of horizontal resolution or the number of forecast members. MOGREPS-G has a 33km horizontal resolution at mid-latitudes and represents temperature characteristics over a large area. MOGREPS-UK would be better at representing sub-regional scale temperature variations, but this currently only has a forecast lead time of 36 hours.

## Discussion

A proof-of-concept for a potential new method for alerting professionals and the public to adverse temperatures in England in order for them to take actions to protect health has been investigated. Its advantages and shortcomings have been tested via a case-study approach, considering one summer and one winter episode of adverse temperatures. By comparing its performance with that of the current system, this prototype has shown some clear advantages over the existing system.

Firstly, the design of the prototype links the first guess alerts to defined impacts on mortality, rather than using the normal distribution of seasonal temperatures to identify triggers. This may be easier to communicate and may be more likely to encourage action by the end-user. Furthermore, it allows a graded increase in response as opposed to the on-off approach of the current system, reflecting the gradual change in health impacts as outdoor temperatures increase or decrease, with the green level corresponding to the current level 1 ‘seasonal preparedness and action’. It has the added advantage of not restricting alerts to the seasons currently used by the HWP and CWP (Jun-Sep and Nov-Mar respectively) so ensuring action to protect health is triggered when hot or cold days occur outside of these months.

Secondly, the prototype links a unique range in relative risk of death to a different temperature range for each region allowing a much more detailed and traceable view of the first guess alerts which are generated (see bottom left panels in Fig 2 and Fig 3). Therefore, the end user is presented with both a national summary of the alerts, a regional level alert and a more detailed local picture. The latter in particular can show more than one alert level over a given region for the same day, while the national summary assigns a single alert level per region. There have been ongoing discussions on the value that such finer information could bring to the end users and local authorities. The main feedback received so far has clearly shown an interest in adopting such an approach (e.g. [19]).

Thirdly, as demonstrated by the summer case study, the design allows alerts to be issued at an earlier stage, providing Public Health England and other organisations and the front-line staff caring for vulnerable people with an extended time-frame to implement action plans. The confirmation/upgrade of the alert level as time progresses is also potentially very useful for a decision making process. Note that this could have also worked in the opposite direction; an early alert is always given a relatively low confidence and therefore a low level warning, despite its possible high impact. If this is issued but not confirmed at later stages, the level can be downgraded accordingly.

Lastly, we have demonstrated that alerts for adverse temperatures could be framed in terms of likelihood and impact, as used by the UK National Severe Weather Warning Service for other weather hazards, which may bring benefits in terms of a more unified system for weather alerts for end-users.

There are, however, some important considerations to be examined before such a system could be recommended for use. Work is first required to objectively verify the methodology (e.g. by applying the approach to several winter and summer seasons and testing forecasts with end-users). The impact thresholds used in this prototype, point to high alert frequencies. We will need to explore where to set the thresholds to produce yellow, amber and red alerts for both heat and cold, in order to provide the best balance of alerting professionals and the public to the actions needed, avoiding ‘alert fatigue’ and the resultant lack of action, and maintaining consistency with the NSWWS. Initial experience with the prototype first-guess alert tool suggests higher thresholds of RR may be appropriate especially in summer. Such decisions will need consultation with a wide range of stakeholders. Appropriate actions will need to be identified at each level of alert for responders and the public, and the professional/public facing final versions will need to be presented in a simple and clear way to ensure the new system is easy to understand by all. Within this context the feedback from the stakeholders will be crucial, particularly the usage of the “two-layer information” whereby the end user is presented at first with the national map of warnings divided by region, and–if need be—the possibility to zoom-in over any given region to explore the geographical pattern of the alert levels (as shown in Figs 2 and 3).

## Conclusions

The links between public health and meteorological services warnings are key for understanding the future risks from extreme temperature events both here in England but also more widely in Europe and internationally. In the IPCC Assessment Report 5 [30] it states that “until mid-century, projected climate change will impact human health mainly by exacerbating health problems that already exist and throughout the 21st century, climate change is expected to lead to increases in ill-health in many regions and especially in developing countries with low income, as compared to a baseline without climate change. Examples include greater likelihood of injury, disease, and death due to more intense heat waves” This paper reports a way forward on that close collaboration on the evidence base science to provide best advice across disciplines can be shared to facilitate better outcomes for populations at risk [31].

A prototype of a potential new alert system to protect the population’s health from adverse temperatures has been developed based on *i)* an impact measure which draws consistently from epidemiological evidence and *ii)* a measure for the confidence of the impact forecast. It also meets the objectives of consistency with the NSWWS and a more refined local approach to alerts. First guess alerts can be generated with a greater lead-in time than the existing system. Once an alert is issued, it is possible to either up- or downgrade the alert level over time as a function of an increasing (decreasing) RR of death and/or a higher (lower) confidence of the given forecast.

The implementation of an impact axis tailored according to regional epidemiological data makes the system extremely flexible. The temperature-mortality relationship is not static and will likely vary depending on climate change and the human adaptation to it. This implies regular reviews of the system, incorporating the most recent epidemiological data available, but little effort will be required to update the system with the new information, and no change to the graphical interface and the way the end-user engages with it.

For operational implementation, further testing alongside the existing warning system will be required. It will also be necessary to garner the views of stakeholders and end-users on the usefulness of the proposed system and the appropriate impact thresholds to be used in the system.

## Supporting Information

S1 Table Lists of potential end-users of the new system and their primary point of contact for extreme weather alerts. (DOCX)
