# Supplementary material for: Improving the Health Forecasting Alert System for Cold Weather and Heat-Waves In England: A Proof-of-Concept Using Temperature-Mortality Relationships
Source: PLoS One. 2015 Oct 2;10(10):e0137804. doi: 10.1371/journal.pone.0137804 (PMC4592211; doi:10.1371/journal.pone.0137804)
Supplement: S1 Table — (DOCX) [file pone.0137804.s001.docx]

Supplementary Material

| Primary point of contact | End-User | Heatwave alerts | Cold Weather alerts |
| --- | --- | --- | --- |
| Local authorities | Social services | ✓ | ✓ |
|  | Schools | ✓ | ✓ |
|  | Residential Homes | ✓ | ✓ |
|  | Nurseries and kindergartens | ✓ | ✓ |
|  | Voluntary organisations | ✓ | ✓ |
|  | Day care centres | ✓ | ✓ |
|  | Winter warmth advice centres |  | ✓ |
| National Health Service England | NHS Choices | ✓ | ✓ |
|  | Ambulance Trusts | ✓ | ✓ |
|  | Pharmacies | ✓ | ✓ |
|  | General Practitioners and District Nurses | ✓ | ✓ |
|  | Hospital trusts | ✓ | ✓ |
|  | Walk-in centres | ✓ | ✓ |
|  | Community health service providers | ✓ | ✓ |
|  | Mental health trusts | ✓ | ✓ |
|  | Care and nursing homes | ✓ | ✓ |
| Media (TV, Radio, Newspapers) | Public | ✓ | ✓ |
| Civil contingencies secretariat and other government departments and agencies | Prison service | ✓ | ✓ |
|  | Winter resilience network |  | ✓ |
|  | Summer resilience network | ✓ |  |

S1 Table. Lists of potential end-users of the new system and their primary point of contact for extreme weather alerts.
